# Multiparametric MRI to stratify risk factors for hemorrhagic complications in inoperable glioblastomas following stereotactic needle biopsy

**DOI:** 10.1007/s00234-025-03769-w

**Published:** 2025-09-16

**Authors:** Matia Martucci, Claudia Tocilă-Mătășel, Luigi Ruscelli, Giuseppe Varcasia, Giammaria Marziali, Francesco Schimperna, Giovanni Pentassuglia, Amato Infante, Quintino Giorgio D’Alessandris, Alessandro Olivi, Simona Gaudino

**Affiliations:** 1 Advanced Radiology Center (ARC), Department of Oncological Radiotherapy, and Hematology, Fondazione Policlinico Universitario Agostino Gemelli IRCCS, Rome, Italy; 2 Iuliu Hațieganu University of Medicine and Pharmacy, Cluj-Napoca, Romania; 3 Department of Neuroradiology, ASST Papa Giovanni XXIII, Bergamo, Italy; 4 Catholic University of the Sacred Heart, Rome, Italy; 5 Department of Neurosurgery, Fondazione Policlinico Universitario Agostino Gemelli IRCCS, Rome, Italy

**Keywords:** Brain tumor, Glioblastoma, Brain biopsy, MRI, Hemorrhage

## Abstract

**Purpose:**

Histological confirmation of glioblastoma (GB) is essential for therapeutic planning, even in inoperable cases where stereotactic needle biopsy (STNB) is the only option. However, post-procedural bleeding remains a known risk. This study aimed to evaluate the association between MRI features of GB and hemorrhagic complications following STNB.

**Methods:**

This retrospective, single-center study included 78 patients with IDH-wildtype GB (mean age: 61 years; 33 females) who underwent pre-biopsy MRI (including SWI and DSC-perfusion) and post-biopsy CT within 72 h. Lesions were anatomically classified into four groups based on their location: cortical/superficial grey matter (sGM *n* = 12), subependymal white matter (sWM; *n* = 36), deep nuclei/thalamus (*n* = 26), or brainstem (*n* = 4). Hemorrhage incidence and area were correlated with lesion location, intratumoral susceptibility signal (ITSS) grade, rCBVmax values, and peritumoral edema. Clinical outcomes were also recorded.

**Results:**

Hemorrhage incidence significantly differed by lesion location (*p* = 0.009), with the highest frequency in deep lesions (85%). Most non-hemorrhagic cases (53%) occurred in sWM. While rCBVmax did not correlate with hemorrhage incidence, a significant linear association with hemorrhage area was noted (*p* = 0.016, *r* = 0.331). Grade 3 ITSS lesions showed more extensive bleeding. No correlation was found between peritumoral edema and bleeding. Most hemorrhages were asymptomatic; only two patients experienced transient neurological symptoms.

**Conclusions:**

Lesion location was the strongest predictor of post-biopsy hemorrhage. The absence of correlation between rCBVmax and bleeding risk suggests biopsies can be safely performed even in hyperperfused (and potentially more aggressive) tumor areas. STNB remains a safe and valuable diagnostic tool when appropriate preoperative evaluation and postoperative monitoring are ensured.

## Introduction

Brain tumors represent a diagnostic and therapeutic challenge due to their variability, the complexities associated with surgery (difficulty in achieving radical resection, involvement of eloquent areas, etc.) and the high prevalence of aggressive and recurrent subtypes. Despite the availability of multiple therapeutic options, a comprehensive understanding of the tumor’s histological subtype and molecular profile stands as the cornerstone of effective treatment strategies. Consequently, even in cases deemed inoperable, obtaining a biopsy sample is essential for diagnosis and personalized treatment planning [1]. Stereotactic needle biopsy (STNB) is a widely used technique for obtaining tissue samples from inoperable or deep-seated brain tumors, allowing detailed histological analysis with a relatively less invasive approach compared to total resection [2, 3]. However, this procedure is not without risks. One of the main complications associated with brain biopsies is intracranial hemorrhage, a potentially severe event that can significantly affect postoperative morbidity and mortality [4–6]. The reported rate of intracranial hemorrhage after stereotactic biopsy ranges from 0 to 11.5% [7]; notably, in a previous work from our group on 93 brain biopsies, we reported no cases of such complication [8]. However, these figures refer to symptomatic hemorrhages; it can be argued that the detection rate of any hemorrhagic foci at post-operative CT is much higher, although not clinically relevant. Several factors have been associated with an increased risk of hemorrhagic complications following biopsy, both related to patients (such as anticoagulant or antiplatelet use) or imaging features [9–12]. However, the current literature on this topic is fragmented and heterogeneous, making it difficult to draw definitive conclusions that can reliably guide clinical/technical decisions. Available data often include different tumor types, biopsy techniques, and a broad spectrum of imaging features, without systematic, clinically applicable insights [13]. Our study aims to systematically investigate the association between magnetic resonance (MR) characteristics of glioblastoma, the most common malignant brain tumor, and the risk of hemorrhage following stereotactic needle biopsy (STNB).

## Methods

### Study population

We retrospectively collected data on consecutive patients with brain tumors who underwent brain tumor biopsy at our institution, from July 2014 to September 2023.

We enrolled all patients with pathologically confirmed glioblastoma, IDH-wildtype (WHO grade IV), who had undergone pre-biopsy MRI scans, including dynamic susceptibility contrast (DSC) perfusion and susceptibility-weighted imaging (SWI) sequences, as well as a computed tomography (CT) scan performed within the initial 72 h following the biopsy procedure.

All biopsies were performed using a stereotactic frameless technique on a StealthStation surgical consolle (Medtronic, Minneapolis, USA), as previously described [8]. The indication for biopsy was primarily based on lesion eloquence and, in selected cases, multicentricity, rather than the patient’s performance status. All cases were discussed within a multidisciplinary tumor board to ensure appropriate clinical decision-making. In our clinical practice, we consistently use a single needle trajectory with three to four intratumoral samples taken in different directions, each within a maximum distance of 1 mm. Intraoperative frozen section analysis is not routinely used in our center.

This study was approved by the Internal Clinical Research Review Board (approval number: Prot N 0019193/24, dated 25/07/2024), and due to its retrospective nature, the requirement for informed consent was waived. All clinical information was handled anonymously.

### Imaging data

Pre-biopsy MR scans were evaluated by two neuroradiologists in consensus to define tumor location, intra-tumoral susceptibility signal score on SWI (ITSS), maximum relative cerebral blood volume (rCBVmax) at DSC-PWI and peritumoral edema.

**Fig. 1:**
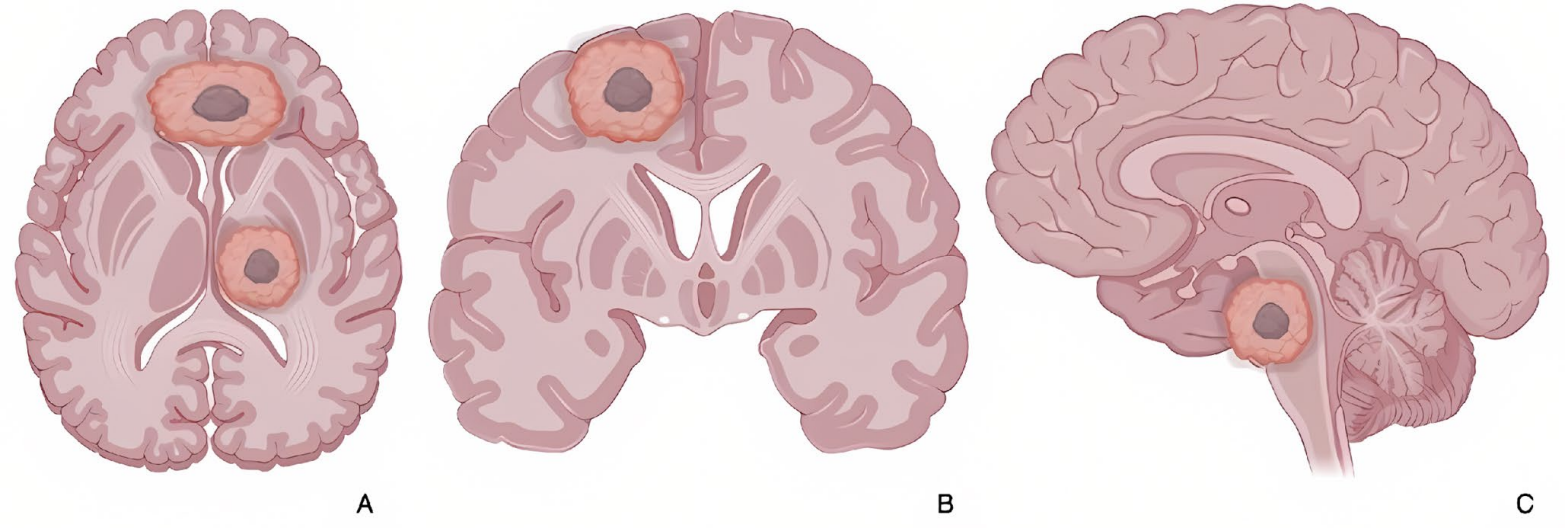
Schematic representation of the 4 GB group locations: in **A**, lesions located in the left thalamus (deep) and within the white matter abating the brain’s ventricles (sWM); in **B** a lesion in the cortical surface of the right frontal lobe (sGM); in **C** a lesion in the pons (brainstem)

Lesions were anatomically divided into four groups based on their location: deep (centered in the basal ganglia and/or thalami), cortical/superficial grey matter (sGM, if centered on the cortical surface of one hemisphere), subependymal white matter (sWM, if the lesion abuts or involves the brain’s ventricles), and brainstem (Fig. 1).

ITSS score was assigned to each lesion as previously reported by Park et al. (0 for no signs of bleeding, 1 for 1–5 dots or intralesional tubular structures, 2 for 6–10 dots or tubular structures, and 3 for more than 10 dots or intralesional tubular structures) [14].

To determine the rCBVmax, three regions of interest (ROIs) were drawn in the solid areas of the lesion showing the maximal CBV values on color map (visually assessed). Then, the ROI showing the highest CBV was selected as the maximum CBV (CBVmax) of the lesion and normalized to the CBV derived from the contralateral normal-appearing white matter (NAWM) to obtain the rCBVmax. Contralateral middle cerebellar peduncle was used in cases of brainstem lesions.

Qualitative assessment of peritumoral edema was also performed, classified as absent, poor, or moderate/severe.

Post-biopsy CT scans were evaluated to detect any new postoperative hemorrhages. The presence and dimensions of the bleeding were recorded. Hemorrhage was assessed at its point of maximum size in axial images. The two largest axial perpendicular diameters were measured, and from these, the hemorrhage area at the point of maximum size was calculated and recorded in square millimeters (mm²).

### Statistical analysis

The population and its clinical and demographic characteristics were described with descriptive statistics techniques: categorical variables are expressed as absolute frequencies and percentages; where necessary, relative frequencies and percentages were used. Quantitative variables are summarized with mean and standard deviation. Normality was checked with the Kolmogorov-Smirnov test. Chi-squared and parametric/non-parametric tests were applied according to quantitative variables normal/non-normal distribution. Student’s t test was applied for continuous variables analysis in paired samples. 1-way ANOVA was performed to compare the intergroup and intragroup variability. Data were analyzed with dedicated software (SPSS for Windows, version 25.0; IBM, Chicago, IL, USA). The data were considered statistically significant at P < = 0.05.

## Results

### Clinical data and outcome

**Fig. 2:**
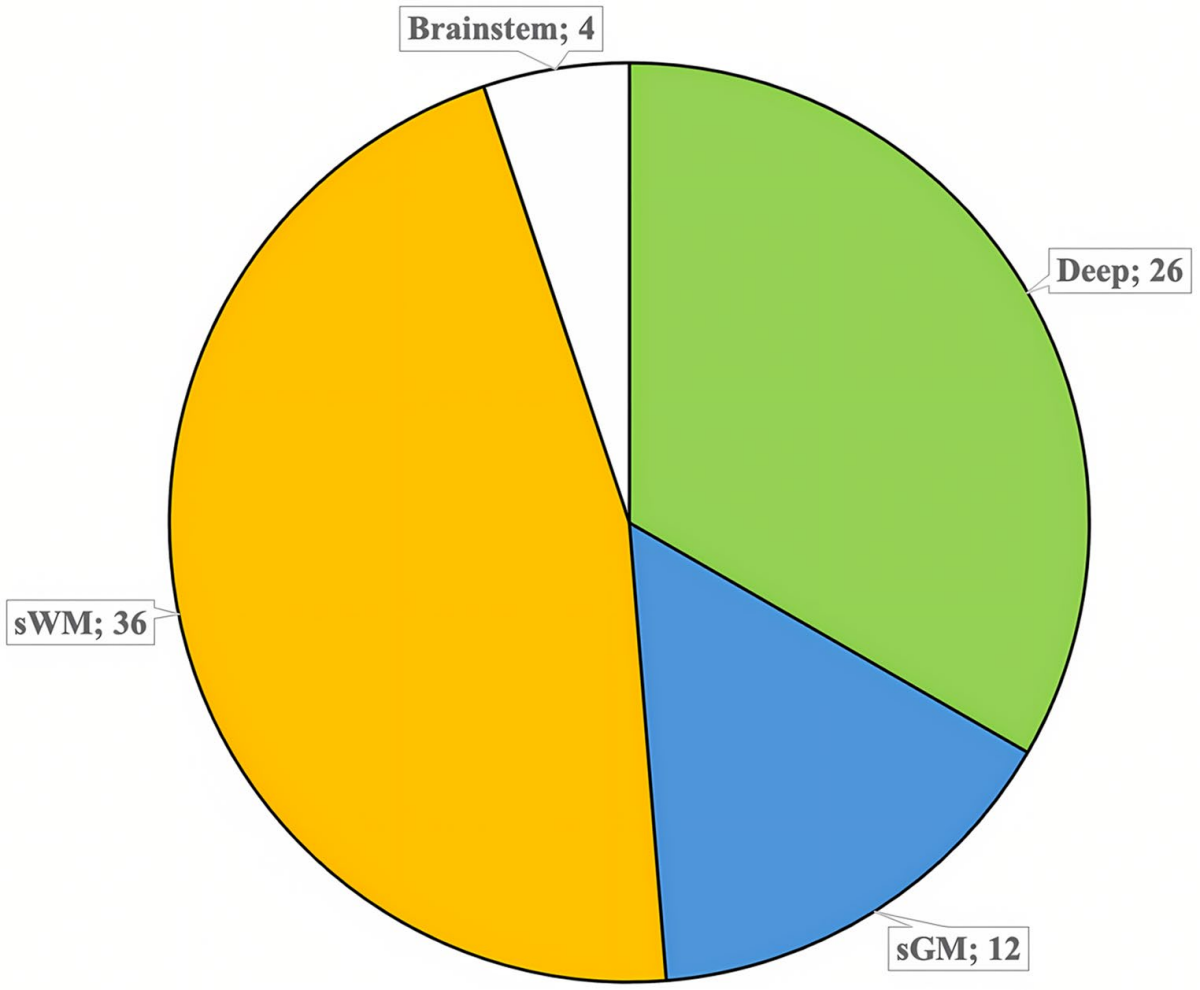
Distribution of lesions by location

Out of 115 patients, 78 (33 Females and 45 Males), with a mean age of 61 years (range 44–80 years) remained eligible for inclusion. Lesions distribution across different locations is detailed in Fig. 2 (12 in sGM, 36 in sWM, 26 in deep regions, 4 in the brainstem).

Median preoperative Karnofsky performance status (KPS) score was 80 (range, 50–100), with only 3 patients having KPS 50. No major bleeding disorders were registered among patients.

Histological diagnosis was successfully achieved in all cases, including those with post-biopsy hemorrhage.

As detailed in Table 1, symptomatic post-biopsies bleeding was reported in 4 (5.1%) patients, aligning with literature data [15]. Of these, two were major bleedings, resulting in death and severe disability, respectively, while the other two caused minor neurological deficits which did not impact on disease treatment. A patient in which biopsy was uneventful, but had to resume anticoagulation for atrial fibrillation, experienced a subdural hematoma 7 days after surgery. Of the two patients with neurological decline unrelated to bleeding, one reported massive brain edema post-biopsy leading to death, and the other was a brainstem tumor patient who had mild facial weakness and diplopia after biopsy, without any impact on postoperative treatments. Overall, an adjuvant therapy after biopsy was performed on 71% patients in the present series.

### Risk of bleeding

**Fig. 3:**
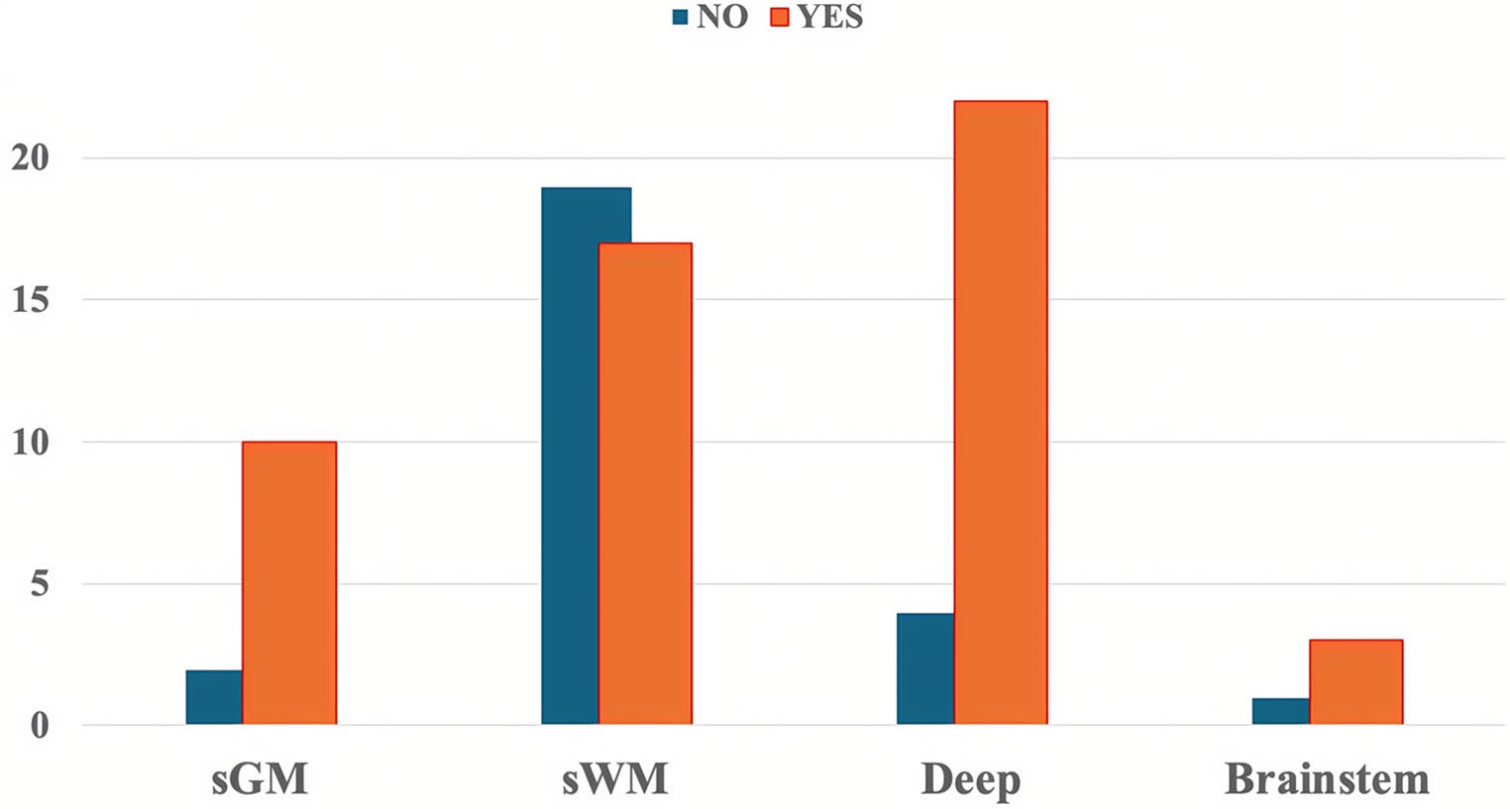
Incidence of post-biopsy hemorrhage in the different locations

Among the patients, 52 out of 78 (67%) experienced post-biopsy bleeding of any size. Significant regional variations in post-biopsy hemorrhage rates were observed across different brain locations (p-value 0.009). The highest bleeding rates were found in deep-seated lesions (22/26, 85%), followed by sGM (10/12, 83%) and brainstem (3/4, 75%). The majority of non-bleeding lesions were located in sWM (19/36, 53%) (Fig. 3).

No correlation was found between ITSS score and bleeding, nor between rCBVmax andbleeding.

No significant differences in ITSS (p 0.16) and rCBVmax (p 0.15) values were observed among different locations.

**Fig. 4:**
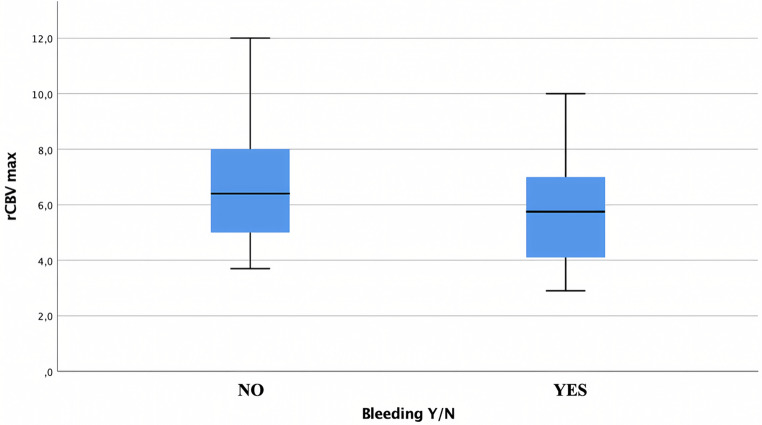
Box plot showing mean rCBVmax in bleeding and non-bleeding lesions

The mean rCBVmax of non-bleeding lesions was 6.5 (+/- 2), similar to that of bleeding lesions at 5.7 (+/- 1.8) (Fig. 4). Bleeding was observed in 77% of lesions with ITSS score 0, 67% with ITSS 1, 55% with ITSS 2, and 61% with ITSS 3, without statistically significant differences (*p* > 0.05).

The extent of peritumoral edema did not show correlation with bleeding.

**Table 1:** Clinical outcome

Parameter	Value
*n*	**78**
Preop KPS, median (range)	80 (50–100)
Postop KPS, median (range)	70 (0-100)
Complications	
Symptomatic bleeding	4 (5.1%)
Late bleeding due to anticoagulation	1 (1.3%)
Neurological deficits unrelated to bleeding	2 (2.6%)
Deaths	3 (3.8%)
Postoperative adjuvant therapy	52/73* (71.2%)

*5 patients were lost at follow-up

### Bleeding area

**Fig. 5:**
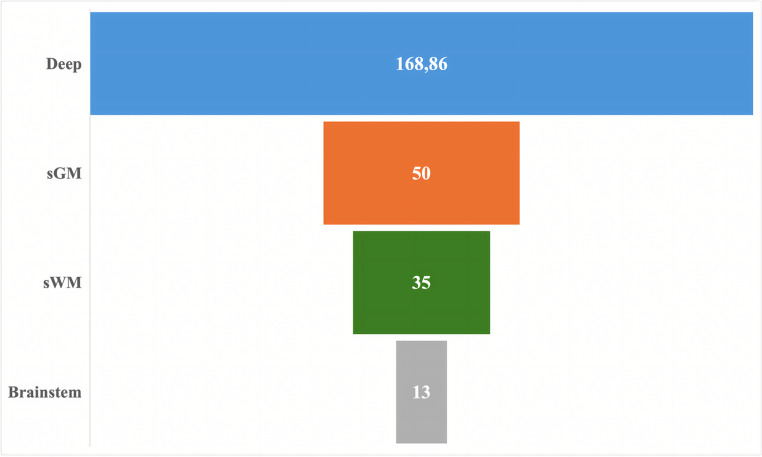
Mean bleeding area in different locations (mm²)

The mean bleeding area was notably higher in deep lesions (168.86 +/- 364.244), though not significantly different compared to other locations (Fig. 5; Table 2).

**Fig. 6:**
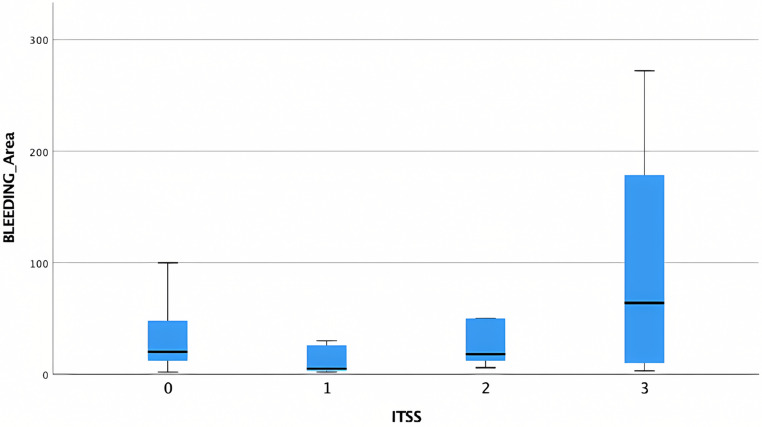
Box plot showing the distribution of mean bleeding areas across different ITSS scores. No significant differences were observed among the different ITSSscores

Lesions with ITSS score of 3 exhibited a higher bleeding area compared to lesions with other ITSS scores (Fig. 6).

**Fig. 7:**
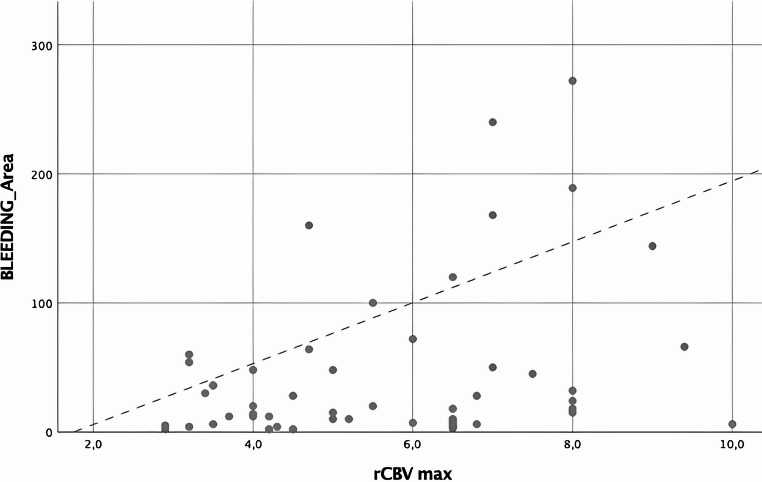
Scatter plot showing the correlation between rCBVmax and bleeding area

A linear correlation was found between rCBVmax and bleeding area (Fig. 7, *p* = 0.016, *r* = 0.331).

**Table 2:** Mean bleeding area in different locations (mm²)

	Mean	std. Dev.	std. Error	95% mean CI		Min	Max
sGM	50	71,56	22,629	−1,19	101,19	2	240
sWM	35	48,808	11,838	9,91	60,09	3	189
Deep	168,86	364,244	77,657	7,37	330,36	2	1440
Brainstem	13	6,557	3,786	−3,29	29,29	7	20

## Discussion

Our study clearly demonstrates that lesion location is an independent risk factor for post biopsy bleeding in inoperable GB. We found deep-seated tumors (centered in the basal ganglia and/or thalami) showing the highest rates of hemorrhage (85%). Also the bleeding area resulted higher in deep lesions compared to other groups. These findings align with prior studies indicating that biopsies in eloquent or deep brain areas are inherently riskier due to the complexity of the procedure and the vulnerability of surrounding vasculature [6, 9]. However, this is the first study including only one tumor type (GB), thus eliminating the confounding bias of other tumor types which can have different bleeding trend or different internal structures that can influence the risk of biopsy bleeding.

**Fig. 8:**
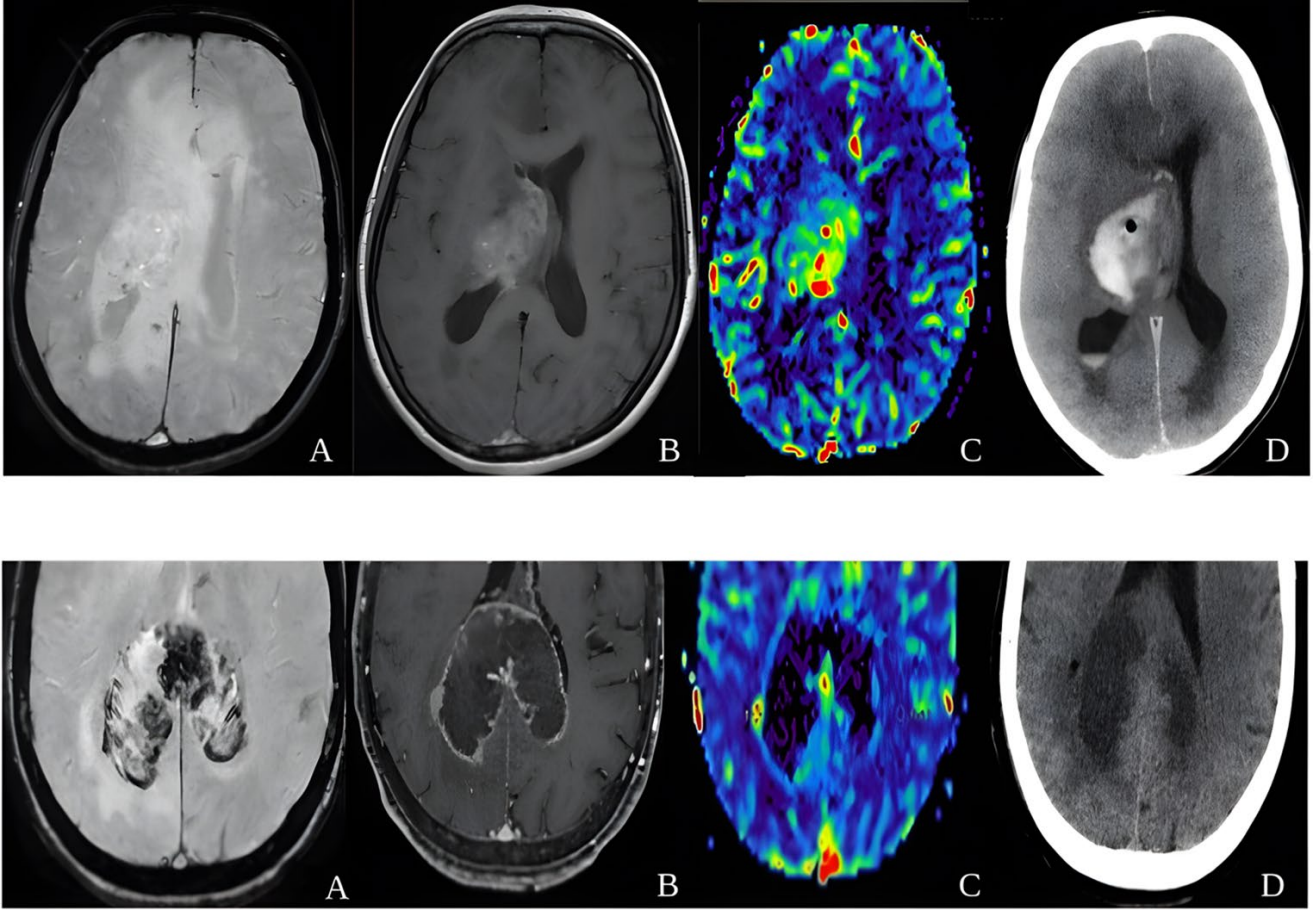
Upper row: (**A**) SWI; (**B**) T1w FSE post-Gad; (**C**) DSC-PWI CBV map; (**D**) CT post-biopsy. Deep-seated GB with ITSS score = 0 (A) and large post-biopsy hemorrhage (D). Lower row: (**E**) SWI; (**F**) 3D-T1w FSPGR post-Gad; (**G**) DSC-PWI CBV map; (**H**) CT post-biopsy. Butterfly GB in the splenium of corpus callosum (sWM), with ITSS score = 3 (E), some hotspot of high rCBV (G), without hemorrhage after biopsy (H)

Deep-seated GB actually bleed more frequent and more conspicuous (Fig. 8). Indeed, neurosurgeon must be aware of this possible complication in such scenario, and consequently clearly inform patients, always ask for post-biopsy CT control and follow-up.

Interestingly, our data did not establish a significant correlation between imaging parameters such as the intratumoral susceptibility signal (ITSS), relative cerebral blood volume (rCBVmax) and peritumoral edema with hemorrhage. The absence of correlation between rCBVmax and bleeding risk has important implications for surgical planning. Specifically, it justifies targeting highly perfused regions for biopsy, as these areas are more likely to represent the most malignant portions of the tumor. By prioritizing biopsies in these regions, clinicians can obtain the most diagnostically and prognostically valuable histological information while optimizing treatment strategies. Such an approach supports personalized therapeutic planning and ensures that critical molecular and histopathological details are not missed [15, 16]. The observed trend of higher hemorrhage areas in lesions with ITSS grade 3 supports the notion that intratumoral vascularity and microbleeds might play a role in predicting hemorrhage extent. However, the lack of statistical significance in our findings might be attributed to the relatively small sample size or the inherent variability in imaging acquisition and interpretation [4, 10].

In our cohort, edema was not found to be associated with an increased risk of bleeding. Although perilesional edema is known to be indicative of altered blood brain barrier and thus of increased vascular “fragility,” data regarding the association between edema and post-biopsy hemorrhages are sparse and controversial [4, 17–19].

Our findings also highlight the potential for postoperative imaging as a critical component of patient management following STNB, particularly for deep-seated GB. Early identification of bleeding can guide timely interventions, mitigating the impact of hemorrhagic complications on patient outcomes [10, 20]. Future prospective studies with larger cohorts and standardized imaging protocols are warranted to elucidate the role of advanced MRI characteristics in predicting hemorrhage and to develop robust, clinically applicable risk stratification tools.

## Conclusion

This study reinforces the importance of lesion location as a determinant of hemorrhagic risk in GB patients undergoing stereotactic needle biopsy. The significant bleeding risks associated with deep-seated tumors call for meticulous pre-procedural planning, careful patient selection, and postoperative monitoring to minimize complications and improve clinical outcomes. Notably, the lack of correlation between rCBVmax and hemorrhage risk supports targeting highly perfused areas for biopsy to maximize diagnostic yield and guide personalized treatment strategies.

## Data Availability

No datasets were generated or analysed during the current study.
